# Common Defects of Spine Dynamics and Circuit Function in Neurodevelopmental Disorders: A Systematic Review of Findings From *in Vivo* Optical Imaging of Mouse Models

**DOI:** 10.3389/fnins.2018.00412

**Published:** 2018-06-19

**Authors:** Nobuhiro Nakai, Toru Takumi, Junichi Nakai, Masaaki Sato

**Affiliations:** ^1^RIKEN Center for Brain Science, Wako, Japan; ^2^RIKEN Center for Advanced Intelligence Project, Tokyo, Japan; ^3^Graduate School of Science and Engineering, Saitama University, Saitama, Japan; ^4^Brain and Body System Science Institute, Saitama University, Saitama, Japan

**Keywords:** two-photon imaging, calcium imaging, autism spectrum disorders (ASDs), dendritic spines, excitatory-inhibitory balance, serotonin

## Abstract

*In vivo* optical imaging is a powerful tool for revealing brain structure and function at both the circuit and cellular levels. Here, we provide a systematic review of findings obtained from *in vivo* imaging studies of mouse models of neurodevelopmental disorders, including the monogenic disorders fragile X syndrome, Rett syndrome, and Angelman syndrome, which are caused by genetic abnormalities of *FMR1, MECP2*, and *UBE3A*, as well as disorders caused by copy number variations (15q11-13 duplication and 22q11.2 deletion) and BTBR mice as an inbred strain model of autism spectrum disorder (ASD). Most studies visualize the structural and functional responsiveness of cerebral cortical neurons to sensory stimuli and the developmental and experience-dependent changes in these responses as a model of brain functions affected by these disorders. The optical imaging techniques include two-photon microscopy of fluorescently labeled dendritic spines or neurons loaded with fluorescent calcium indicators and macroscopic imaging of cortical activity using calcium indicators, voltage-sensitive dyes or intrinsic optical signals. Studies have revealed alterations in the density, stability, and turnover of dendritic spines, aberrant cortical sensory responses, impaired inhibitory function, and concomitant failure of circuit maturation as common causes for neurological deficits. Mechanistic hypotheses derived from *in vivo* imaging also provide new directions for therapeutic interventions. For instance, it was recently demonstrated that early postnatal administration of a selective serotonin reuptake inhibitor (SSRI) restores impaired cortical inhibitory function and ameliorates the aberrant social behaviors in a mouse model of ASD. We discuss the potential use of SSRIs for treating ASDs in light of these findings.

## Introduction

Neurodevelopmental disorders (NDDs), such as autism spectrum disorders (ASDs) and other genetic syndromes, are an etiologically heterogeneous group of neuropsychiatric conditions that manifest very early in life due to the rapid development of brain circuitry during this period (Rutter et al., 2008). While demonstrating many specific symptoms, these disorders often share common deficits such as intellectual disability, epilepsy, sleep disturbances, and abnormal sensory processing. Despite these pronounced deficits, gross brain anatomy often appears largely normal, suggesting that the abnormalities result from relatively subtle changes in connectivity and communication among neurons. The pathogenesis of these disorders thus should be sought at the level of neural circuits and, more specifically, in how neural circuits are initially constructed during development, subsequently refined by experience, and operate when they subserve various cognitive and motor functions. Proper formation, stabilization, function, and remodeling of synapses, each of which is achieved by complex molecular machineries, are essential to ensure the function of these processes.

Neurodevelopmental disorders are caused by various genetic abnormalities and non-genetic factors, such as exposure to toxins and pathogens. To facilitate research, many mouse models that recapitulate the genetic abnormalities known to cause relevant disorders in humans have been established. These genetic mouse models often display phenotypes similar to the symptoms found in individuals with the relevant disorders. Discovering potential targets for effective therapies requires a thorough understanding of these disorders at the molecular, cellular, and neuronal network levels. However, methodologies used to examine the structure and function of the human brain, such as magnetic resonance imaging (MRI), electroencephalography (EEG), and sensory-evoked potentials, do not yet have sufficient spatiotemporal resolution or specificity to distinguish the underlying abnormalities at the cellular level. Alternatively, *in vivo* optical imaging using various microscopic techniques combined with different labeling methods in mice can visualize circuit structure and function in living animals, with cellular and subcellular resolution. This technology is thus well suited for studies that aim to reveal basic pathogenic mechanisms and guide clinical research. In fact, these techniques have been applied successfully to investigate structural and functional abnormalities in several mouse models of NDDs. In this review, we discuss the findings obtained by these studies and explore their implications for a better understanding of the corresponding human disorders.

## Methodologies for *In Vivo* Optical Imaging

**FIGURE 1:**
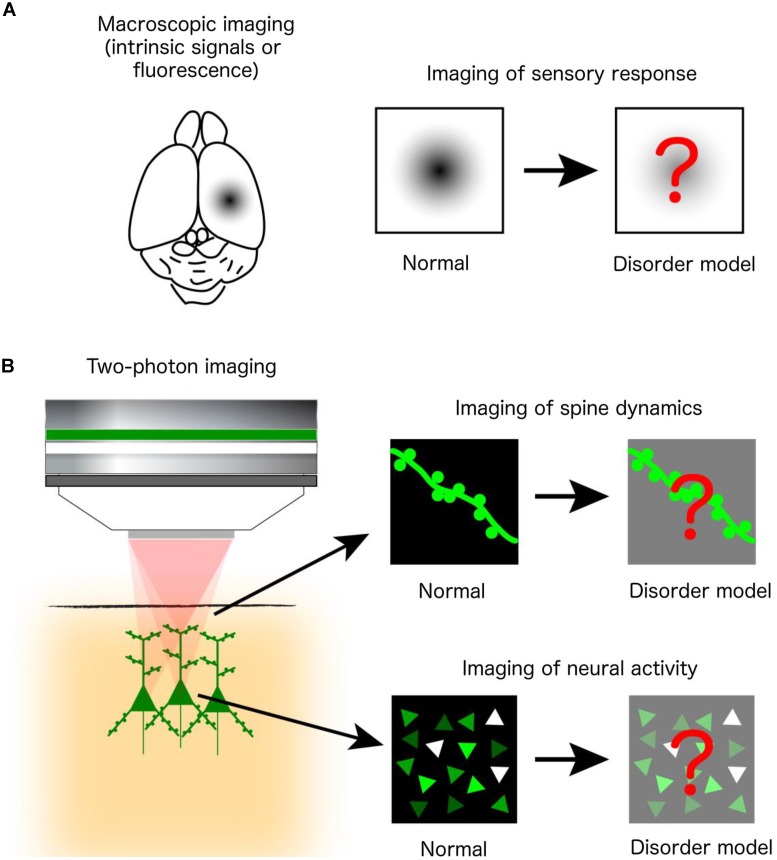
*In vivo* optical imaging in mouse models of NDDs. **(A)** Macroscopic imaging of cortical activity visualizes intrinsic optical signals from brain tissues or the fluorescence of exogenously introduced calcium or voltage indicators, all of which reflect localized brain activity. Studies are designed to identify abnormalities of cortical sensory responses in mouse models of NDDs compared to normal mice. **(B)** Two-photon microscopy at subcellular and cellular resolution images the morphological dynamics of dendritic spines labeled by fluorescent proteins (upper right) or the activity of a population of neurons labeled by fluorescent calcium indicators (lower right).

Human postmortem studies have revealed changes in the density and morphology of dendritic spines, while electrophysiological, psychophysical and neuroimaging analyses indicate abnormal sensory processing and dysfunctional activity in a variety of otherwise etiologically distinct NDDs. However, postmortem studies are limited by uncontrolled tissue changes after death and cannot elucidate changes in spine dynamics, while the aforementioned diagnostic techniques lack the resolution to reveal changes at the single-cell and microcircuit levels. Thus, many *in vivo* optical imaging studies of mouse models have examined the structural dynamics of dendritic spines on cortical neurons and the responsiveness of cortical neuronal populations to sensory stimuli. These experiments are often conducted by acquiring images of the same set of fluorescently labeled spines over time, using two-photon microscopy. Alternatively, circuit function can be revealed by imaging changes in signals of fluorescent indicators of neural activity (e.g., calcium- or voltage-sensitive indicators) or intrinsic optical signals from brain tissues during sensory stimulation (**Figure 1**).

Dendritic spines are tiny (∼1 μm) protrusions on dendrites on which synapses form, primarily excitatory glutamatergic synapses. Historically, the density and shape of spines were examined by Golgi’s impregnation method, which can reveal the detailed morphology of a subset of neurons in fixed brain tissues. However, these images provide only a “snapshot” of highly dynamic spines, so the information provided by these images may be misleading. For example, normal spine density may be a consequence of abnormally enhanced (or reduced) spine formation and concomitantly increased (or decreased) elimination occurring at balanced rates. Thus, *in vivo* time-lapse imaging is a powerful method to determine whether model mice exhibit abnormalities in spine density, shape, and turnover by tracking the fate of individual spines over time. The spines to be imaged *in vivo* are usually labeled with fluorescent marker proteins expressed by means of transfection using viral vectors or *in utero* electroporation (Cruz-Martín et al., 2010; Isshiki et al., 2014) or by crossing the model mouse with a transgenic reporter mouse line (Pan et al., 2010; Landi et al., 2011; Padmashri et al., 2013; Reiner and Dunaevsky, 2015; Kim et al., 2016; Hodges et al., 2017). Labeled spines can then be imaged by two-photon excitation laser scanning fluorescence microscopy, which can efficiently excite fluorescent molecules within otherwise opaque brain tissues via highly penetrating near-infrared pulsed laser light (Denk and Svoboda, 1997). However, the best resolution is still usually obtained from the superficial spines located in layer (L) 1, within ∼100 μm of the cortical surface, which are nonetheless on apical dendrites of pyramidal neurons in L2–5. Imaged spines are classified into different morphological categories, such as thin (immature), stubby, and mushroom (mature), and their appearance, persistence, and disappearance are analyzed in multiple images acquired at intervals of hours, days, and even weeks. In young adolescent mice, over the course of 2 weeks, approximately 10% of spines imaged on L5 pyramidal neurons are eliminated, while 5-8% of them are formed (Zuo et al., 2005). These rates become smaller in adult mice, showing that 3-5% of imaged spines are eliminated or newly formed. Images of spine dynamics have been acquired from multiple cortical areas, including somatosensory (Cruz-Martín et al., 2010; Pan et al., 2010; Landi et al., 2011; Isshiki et al., 2014), visual (Kim et al., 2016), motor (Padmashri et al., 2013; Reiner and Dunaevsky, 2015; Hodges et al., 2017) and frontal (Isshiki et al., 2014) cortices, during area-specific postnatal developmental periods. Spine dynamics in the cortex are known to be influenced by sensory experience and learning through the effects of neuronal activity on molecular machinery in spines, and this reorganization of synaptic connections is thought to underlie adaptive changes in circuit functions (Holtmaat and Svoboda, 2009). The effects of sensory input can be examined through various experimental manipulations, such as whisker trimming to alter the degree and topology of somatosensory input (Pan et al., 2010; Isshiki et al., 2014), visual deprivation by eyelid suture or rearing animals in darkness (Kim et al., 2016), or training mice to acquire new motor skills (Padmashri et al., 2013; Reiner and Dunaevsky, 2015; Hodges et al., 2017).

There are also multiple techniques to image neural circuit activity in the living mouse brain. Intrinsic-signal optical imaging visualizes the area and intensity of cortical activity by extracting optical signals intrinsic to brain tissues, such as subtle changes in red light reflectance, that are correlated with local brain activity (Kalatsky and Stryker, 2003; Tropea et al., 2009; Sato and Stryker, 2010; Arnett et al., 2014; Castro et al., 2014; Gogolla et al., 2014; Banerjee et al., 2016). This technique enables the measurement of sensory-evoked activity in neuronal populations, particularly from superficial cortical layers, without the application of exogenous indicator molecules. However, the temporal resolution of this modality is limited to the order of seconds. In contrast, fast spatiotemporal dynamics of cortical electrical activity can be measured at millisecond resolution with voltage-sensitive dyes (VSDs) that change fluorescence in a membrane potential-dependent manner (Zhang et al., 2014; Connor et al., 2016; Lee L.J. et al., 2017). Alternatively, changes in intracellular calcium ion concentrations associated with neuronal activity can be measured with high spatiotemporal resolution using synthetic calcium-sensitive dyes or genetically encoded calcium indicators (GECIs) introduced into the living brain (Nakai et al., 2001; Looger and Griesbeck, 2012). To label neuronal populations, cell-permeable analogs of synthetic dyes are microinjected locally into the target brain area (Gonçalves et al., 2013; Banerjee et al., 2016). Alternatively, GECIs are expressed by viral vectors (He et al., 2017; Zaremba et al., 2017) or by crossing disorder model mice with transgenic mice expressing the GECI (Nakai et al., 2017). These GECIs are often under the control of promoters specific to certain cell types. *In vivo* calcium imaging can be performed using two-photon microscopy to achieve cellular resolution (Gonçalves et al., 2013; Banerjee et al., 2016; He et al., 2017; Zaremba et al., 2017) or by single-photon excitation macroscopy using charge-coupled device or complementary metal-oxide-semiconductor cameras for wide-field imaging (Nakai et al., 2017). In sum, these techniques allow for a variety of experimental measurements, ranging from imaging spontaneous network activity (Gonçalves et al., 2013; Connor et al., 2016) to sensory encoding (Arnett et al., 2014; Zhang et al., 2014; Banerjee et al., 2016; He et al., 2017; Lee L.J. et al., 2017; Nakai et al., 2017) and neuroplastic changes elicited by altered experience (Tropea et al., 2009; Sato and Stryker, 2010; Castro et al., 2014; Banerjee et al., 2016; Zaremba et al., 2017).

Although it is beyond the scope of this review to discuss all currently available imaging methodologies, *in vivo* brain imaging in mice includes not only optical but also other techniques such as MRI. In particular, resting-state functional MRI (rsfMRI) of the mouse brain is an emerging technique that enables mapping of both local and long-range functional connectivity between distinct brain areas by visualizing anatomical patterns of the low-frequency oscillatory blood-oxygen-level dependent (BOLD) signals associated with spontaneous correlated activity (Gozzi and Schwarz, 2016; Liska and Gozzi, 2016). Although the spatial resolution of this technique is not as high as the above-mentioned optical imaging and its application so far is primarily limited to mapping of intrinsic functional brain networks in anesthetized or sedated animals, it has a great advantage over the optical methods in that it can examine the functional connectivity at the whole-brain scale, including deep subcortical areas that are difficult to access optically. This technique should complement optical methods in future studies such that functional connectivity defects identified by rsfMRI are subsequently examined more closely via two-photon microscopy. A few initial studies using rsfMRI have revealed impaired functional connectivity in mouse models of ASD (Haberl et al., 2015; Sforazzini et al., 2016; Liska et al., 2018).

In the following sections, we review studies on mouse models of NDDs using these *in vivo* optical imaging techniques. Our comprehensive literature searches that covered 35 NDDs (Bishop, 2010) were conducted according to the PRISMA guidelines for systematic reviews (Moher et al., 2009) and identified a total of 22 *in vivo* optical imaging studies in mouse models of fragile X syndrome (FXS), Rett syndrome (RTT), Angelman syndrome (AS), ASDs (including 15q duplication syndrome) and 22q11.2 deletion syndrome (22q11.2DS). The studies selected are summarized in **Table 1**, and the details of the searches are described in the appended footnote.

**Table 1:** *In vivo* optical imaging studies of mouse models of NDDs.

Imaging modality	Disorder	Mouse model	Labeling method	Imaging method	Imaged area	Age	Findings	Reference
Spine	FXS	*Fmr1* KO	*In utero* electroporation	Two-photon	Somatosensory cortex, L2/3 neurons	P7-24	Normal spine density and length. Delayed downregulation of spine turnover and transition from immature to mature spines at P10-12.	Cruz-Martín et al., 2010
	FXS	*Fmr1*^-/y^	Tg mice (YFP-H)	Two-photon	Somatosensory cortex, L5 neurons	3 w.o. -adult	Normal spine density. Enhanced formation and elimination of spines. Increased fraction of transient (immature) spines. Reduced sensitivity of spine formation and elimination to altered sensory experience.	Pan et al., 2010
	FXS	*Fmr1*^-/y^	Tg mice (YFP-H)	Two-photon	Motor cortex, L5 neurons	5 w.o.	Impaired motor learning. Normal density of total spines and filopodia. Enhanced baseline spine formation and elimination. Lack of training-induced increases in spine density and formation.	Padmashri et al., 2013
	FXS	*Fmr1*^-/y^	Tg mice (YFP-H)	Two-photon	Motor cortex, L5 neurons	P35-42	Lack of clustering but normal stabilization of new spines formed after motor skill training.	Reiner and Dunaevsky, 2015
	FXS	Astrocyte-specific *Fmr1*^-/y^	Tg mice (YFP-H)	Two-photon	Motor cortex, L5 neurons	4 w.o. -adult	Normal spine density, normal motor learning, and enhanced basal spine formation in young mice. Increased density of total and thin (immature) spines, impaired motor learning, and lack of enhanced spine formation and elimination during training in adult mice.	Hodges et al., 2017
	FXS	*Fmr1*^-/y^	Tg mice (GFP-M)	Two-photon	Visual cortex, L5 neurons	2-6 m.o.	Enhanced gain and loss of spines. No increased spine turnover was observed in the enriched environment. Rescue of enhanced spine turnover by MMP-9 inhibition.	Nagaoka et al., 2016
	RTT	*Mecp2*^-/*y*^	Tg mice (GFP-M)	Two-photon	Somatosensory cortex, L5 neurons	P25-40	Reduced spine and filopodia density and reduced short-term changes in spine length and head volume at P25–26. Rescue of short-term spine dynamics defects but not reduced spine density by IGF-1. Reduced spine density and normal short-term spine dynamics at P40.	Landi et al., 2011
	AS	*Ube3a*^m-/p+^	Tg mice (GFP-O)	Two-photon	Visual cortex, L5 neurons	P14-38	Decreased spine density. Normal spine formation and increased spine elimination. Increased fraction of thin spines.	Kim et al., 2016
	ASD	15q dup (also Neuroligin-3 R451C)	*In utero* electroporation	Two-photon	Somatosensory cortex and anterior frontal cortex, L2/3 neurons	2-8 w.o.	Normal spine density. Enhanced gain and loss of PSD-95 containing spines. Reduced sensitivity of spine formation to altered sensory experience.	Isshiki et al., 2014
Activity	FXS	*Fmr1* KO	Synthetic calcium indicator injection	Calcium imaging (two-photon)	Somatosensory cortex, L2/3 neurons (anesthetized and awake)	P9-40	Higher synchrony of spontaneous ensemble activity. Higher proportion of neurons participating in the synchrony. Higher synchrony during sleep.	Gonçalves et al., 2013
							Lack of synchrony modulation by anesthesia.	
	FXS	*Fmr1*^-/y^	No labeling	Intrinsic-signal	Somatosensory cortex	12-14 w.o.	Increased area of response to tactile stimulation.	Arnett et al., 2014
				optical imaging	(anesthetized)			
	FXS	*Fmr1*^-/y^	Synthetic VSD application	VSD imaging	A large cortical area including somatosensory and motor cortices (anesthetized)	10-16 w.o.	Accelerated spread of tactile-evoked cortical activity.	Zhang et al., 2014
	FXS	*Fmr1* KO	AAV vector-mediated GCaMP6s expression	Calcium imaging (two-photon)	Somatosensory cortex, L2/3 neurons (awake)	P14-adult	Increased avoidance behavior to tactile stimulation and reduced fraction of cells responding to tactile stimulation in young mice. Impaired neuronal adaptation to repetitive tactile stimulation in young and adult mice.	He et al., 2017
	RTT	*Mecp2*^-/+^	No labeling	Intrinsic-signal optical imaging	Visual cortex (anesthetized)	P28-60	Abnormally prolonged plasticity at P60 in response to altered visual experience and rescue by a tripeptide form of IGF-1 or full-length IGF-1	Tropea et al., 2009; Castro et al., 2014
	RTT	*Mecp2*^-/*y*^ and PV^+^- or SOM^+^-interneuron specific *Mecp2*^-/*y*^	Synthetic calcium indicator injection and no labeling	Calcium imaging (two-photon) and intrinsic-signal optical imaging	Visual cortex (anesthetized and awake), L2/3 neurons	P28-60	Reduced response rate, reliability, selectivity and signal-to-noise ratio of pyramidal neurons to visual stimuli in *Mecp2*^-/*y*^ mice. Recapitulation of visual response defects in PV^+^- but not SOM^+^-specific *Mecp2*^-/*y*^ mice. Improvement of visual response defects by IGF-1. Abnormally prolonged plasticity of PV^+^-specific *Mecp2*^-/*y*^ mice at P60.	Banerjee et al., 2016
	RTT	*Mecp2*^-/*y*^	Synthetic VSD application	VSD imaging	Somatosensory cortex (anesthetized)	1-2 m.o.	Weaker and more diffuse tactile-evoked responses.	Lee L.J. et al., 2017
	AS	*Ube3a*^m-/p+^	No labeling	Intrinsic-signal optical imaging	Visual cortex (binocular zone) (anesthetized)	P21-37	Lack of rapid plasticity in response to altered visual experience in P25-28 mice. Abnormally immature form of plasticity in P33-37 mice.	Sato and Stryker, 2010
	ASD	15q dup	Tg mice (GLT-1-G-CaMP7)	Calcium imaging (macro-scopic)	Somatosensory cortex (anesthetized)	7-8 w.o.	Reduced magnitude, slower decay and broader area of response to tactile stimulation.	Nakai et al., 2017
	ASD	BTBR T+tf/J (also *Shank3* KO and *Mecp2* KO)	No labeling	Intrinsic-signal optical imaging	Insular cortex (anesthetized)	P16-adult	Impaired multisensory integration and its maturation. Increased area of auditory response. Rescue of impaired integration in the adult by enhanced inhibition early in life.	Gogolla et al., 2014
	ASD	*Mdga2*^+/-^	Synthetic VSD application	VSD imaging	Nearly entire dorsal cortex of one hemisphere (anesthetized)	Adult (6-10 w.o.)	Enhanced spontaneous cortical activity in motor and retrosplenial cortices. Functional hyperconnectivity in lateral cortical areas.	Connor et al., 2016
	22q11.2DS	*Df(16)A^+/-^*	AAV vector-mediated GCaMP6f expression	Calcium imaging (two-photon)	Hippocampal CA1 neurons (awake)	8-12 w.o.	Impaired goal-oriented learning. Reduced place cell map stability. Absence of goal-directed reorganization of place cell maps.	Zaremba et al., 2017


*FXS, fragile X syndrome; RTT, Rett syndrome; AS, Angelman syndrome; ASD, autism spectrum disorder; 22q11.2DS, 22q11.2 deletion syndrome; *Fmr1*^-*/y*^, male hemizygous *Fmr1*-null mice; *Mecp2*^-*/y*^, male hemizygous *Mecp2*-null mice; *Mecp2*^-/+^, female heterozygous *Mecp2*-null mice; *Ube3a*^*m-/p*+^, maternal *Ube3a*-deficient mice; 15q dup, paternal 15q duplication mice; *Mdga2*^+/-^, heterozygous *Mdga2*-null mice; *Df(16)A*^+/-^, mice carrying a hemizygous deletion of the syntenic region of mouse chromosome 16; w.o., weeks old; m.o., months old. We comprehensively searched the PubMed database of the U.S. National Library of Medicine (https://www.ncbi.nlm.nih.gov/pubmed/) as of early April 2018 using the keywords “imaging” and “mice” in combination with each of 35 NDDs that were identified from Rutter’s Textbook of Child and Adolescent Psychiatry, 5th Edition by Bishop (Rutter et al., 2008; see Table 1 of Bishop, 2010). The number of studies retrieved for each NDD ranged from 0 to 178. We further selected from these studies the ones that conducted cellular, subcellular or macroscopic-resolution optical imaging of brain function or structure in living animals, after which 8, 3, 2, 2 and 1 remained for mouse models of FXS, RTT, AS, ASD and 22q11.2DS. Finally, we added two FXS studies (Padmashri et al., 2013; Nagaoka et al., 2016), two RTT studies (Tropea et al., 2009; Castro et al., 2014) and two ASD studies (Gogolla et al., 2014; Nakai et al., 2017), which are relevant but did not appear in the initial database searches, probably because some keywords were not included in the searchable part of the literature.*

## *In Vivo* Optical Imaging of FXS Model Mice

Fragile X syndrome is the most common inherited mental impairment and also the most common known single genetic cause of ASD, with a total frequency of approximately 1 in 4,000 males and 1 in 8,000 females (Riley et al., 2017). Individuals with FXS exhibit a broad range of symptoms, including intellectual disabilities, autism, macroorchidism, seizures, sensory hypersensitivity, and facial abnormalities such as a long face and large ears (Garber et al., 2008). In almost all cases, FXS is caused by a CGG repeat expansion in the 5′ untranslated region of the fragile X mental retardation 1 gene (*FMR1*) and hypermethylation of its promoter region on the X chromosome, which results in *FMR1* transcriptional silencing and absence of the fragile X mental retardation protein (FMRP) encoded by this gene (Santoro et al., 2012).

Fragile X mental retardation protein is a polyribosome-associated RNA-binding protein localized in the soma and nucleus as well as the dendrites and spines of neurons (Feng et al., 1997). FMRP is also expressed in astrocytes of the developing brain (Pacey and Doering, 2007). It regulates the trafficking and local translation of mRNAs for numerous genes important for synaptic growth, function, and plasticity (Bagni and Greenough, 2005; Darnell and Klann, 2013; Contractor et al., 2015). *Fmr1* knockout (KO) mice, including the often-used male hemizygous *Fmr1*-null (*Fmr1*^-/y^) mice, lack FMRP expression and recapitulate many behavioral features observed in FXS (Bernardet and Crusio, 2006). Golgi impregnation of cerebral cortical autopsy materials from individuals with FXS has revealed higher spine density and greater numbers of long, thin, immature-looking spines on L3 and L5 pyramidal neurons of parieto-occipital neocortex (Rudelli et al., 1985; Hinton et al., 1991) and on L5 pyramidal neurons of temporal and visual cortices (Irwin et al., 2001). Adult *Fmr1* KO mice exhibited similar morphological defects of spines on visual cortical L5 pyramidal neurons when examined by Golgi staining of fixed tissues (Comery et al., 1997). These spine abnormalities appear to be age-dependent because the aberrantly high spine density and greater spine lengths observed on green fluorescent protein (GFP)-transfected L5 pyramidal neurons of somatosensory barrel cortex during the early postnatal period (1 week of age; Nimchinsky et al., 2001) and on Golgi-stained L5 pyramidal neurons during adulthood (postnatal day 73-75 of age (P73-75); Galvez and Greenough, 2005) were less apparent in juvenile mice at approximately 4 weeks of age (Nimchinsky et al., 2001; Galvez and Greenough, 2005).

*Fmr1* KO mice are among the most extensively studied NDD models, and cortical defects are relatively well described at both the molecular and circuit levels (Contractor et al., 2015). *In vivo* two-photon time-lapse imaging of *Fmr1* KO mice revealed elevated spine turnover (i.e., greater spine formation and elimination) along apical dendritic tufts of L2/3 (Cruz-Martín et al., 2010) and L5 (Pan et al., 2010) pyramidal neurons in the barrel cortex and on L5 pyramidal neurons in the motor cortex (Padmashri et al., 2013) at different ages. Although the overall spine density in these studies appeared surprisingly normal, larger populations of short-lived small spines were observed in *Fmr1* KO mice (Cruz-Martín et al., 2010; Pan et al., 2010). Another study using astrocyte-specific *Fmr1* KO mice reported normal spine density but increased spine formation in young mice as well as higher densities of total and thin spines on the apical dendritic tufts of L5 motor cortex pyramidal neurons in adult mice (Hodges et al., 2017), demonstrating a significant contribution of astrocytic FMRP to the neuronal wiring defects in global *Fmr1* KO mice. Further, these mice demonstrate reduced sensitivity of spine turnover to altered sensory experience (Pan et al., 2010) and motor learning (Padmashri et al., 2013; Hodges et al., 2017). For instance, trimming all whiskers reduced the spine elimination rate of L5 pyramidal neurons in the contralateral barrel cortex in wild-type mice but not in *Fmr1* KO mice, while trimming alternate whiskers in a chessboard-like pattern increased the spine formation rate in wild-type mice but not in *Fmr1* KO mice (Pan et al., 2010). Moreover, training wild-type mice on a forearm-reaching task increased total spine numbers on L5 pyramidal neurons of the motor cortex contralateral to the trained arm through an increase in spine formation rate. Conversely, in *Fmr1* KO mice, motor-skill learning was impaired, and spine number and formation rate were not altered (Padmashri et al., 2013). A follow-up study by the same group reported that the new spines formed after motor skill training in *Fmr1* KO mice lacked clustering on dendrites but exhibited a degree of stabilization similar to that in wild-type mice (Reiner and Dunaevsky, 2015). Impaired motor-skill learning and lack of training-induced increases in spine formation and elimination rates were also observed in adult astrocyte-specific *Fmr1* KO mice trained on a similar task, although learning appeared normal when these mice were trained during adolescence (Hodges et al., 2017).

A recent study reproduced the elevated basal spine turnover and the lack of experience-dependent enhancement in L5 visual cortical pyramidal neurons of *Fmr1* KO mice (Nagaoka et al., 2016) and further reported that the abnormal baseline spine turnover can be rescued by pharmacological inhibition of matrix metalloproteinase-9 (MMP-9). The transport and translation of mRNAs encoding MMP-9 are regulated by FMRP at synapses (Janusz et al., 2013), and pharmacological inhibition and genetic deletion of MMP-9 rescued the dendritic spine and behavioral abnormalities in *Fmr1* KO mice (Bilousova et al., 2009; Sidhu et al., 2014). Moreover, a clinical trial of the antibiotic MMP-9 inhibitor minocycline demonstrated some global benefits to children and adolescents with FXS (Leigh et al., 2013). Together, the findings from these *in vivo* imaging studies demonstrate that the absence of FMRP reduces baseline synaptic stability and impairs experience-dependent and learning-induced spine remodeling in the cortex. These defects support the idea that developing synaptic circuits may not be properly shaped by sensory stimuli and learning in FXS.

Individuals with FXS and *Fmr1* KO mice are also known to display features of hyperexcitability at both neurological and behavioral levels (Bernardet and Crusio, 2006; Garber et al., 2008; Contractor et al., 2015). Functional imaging studies of *Fmr1* KO mice have shed light on potential neural circuit bases for such symptoms. In one study, two-photon calcium imaging of somatosensory cortical networks in early postnatal *Fmr1* KO mice demonstrated greater and more widespread synchrony of spontaneous ensemble activity during both wakefulness and sleep and a delay in the developmental decorrelation of this synchrony (Gonçalves et al., 2013). In adult *Fmr1* KO somatosensory cortex, tactile stimulation evoked larger response regions than in wild-type mice, as revealed by intrinsic-signal optical imaging (Arnett et al., 2014), and accelerated spread of cortical activity, as evidenced by VSD imaging (Zhang et al., 2014). Subsequent electrophysiological recordings revealed that this hyperexcitability can be partly attributed to defects in neuronal dendritic h-channels and BK_Ca_ channels (Zhang et al., 2014). Collectively, these findings suggest that similar circuit and molecular defects in the human brain may underlie FXS symptoms associated with neuronal hyperexcitability, such as hyperarousal, high susceptibility to seizures, and sensory hypersensitivity. A recent two-photon calcium imaging study of somatosensory cortex in head-fixed behaving *Fmr1* KO mice reported that increased avoidance to tactile stimulation, analogous to the FXS symptom known as tactile defensiveness, was associated with a reduced fraction of cells responding to the stimuli in young mice and with impaired neuronal adaptation to repetitive tactile stimulation in young and adult mice (He et al., 2017).

## *In Vivo* Optical Imaging of RTT Model Mice

Rett syndrome is a severe postnatal neurological disorder named after the Austrian pediatrician Andreas Rett, who first described this condition in 1966 (Hagberg et al., 1983). RTT is caused by loss-of-function mutations of the methyl-CpG binding protein 2 gene (*MECP2*), which is located on the X chromosome and encodes a chromatin protein that is involved in epigenetic transcriptional regulation of many genes (Chahrour and Zoghbi, 2007; Lyst and Bird, 2015). MECP2 is expressed broadly throughout the body but most abundantly in mature neurons. RTT is an X-linked dominant disorder and almost exclusively affects females because males hemizygous for *MECP2*-null mutations usually display severe early postnatal encephalopathy and do not survive infancy. RTT affects approximately 1 in 10,000–15,000 girls (Hagberg, 1985), and the vast majority of cases are sporadic. Typical RTT is characterized by apparently normal development during the first 6–18 months of life, followed by a period of regression and then recovery or stabilization of various symptoms, including loss of acquired purposeful hand skills, loss of acquired spoken language, gait abnormalities, and stereotypic hand movements such as wringing, clapping, mouthing and washing (Neul et al., 2010). Individuals with RTT often display a variety of other symptoms, including slowing of the rate of head growth, breathing abnormalities, impaired sleep patterns, and seizures. Some individuals eventually diagnosed with RTT are diagnosed initially with autism, and autistic features such as social withdrawal are more common in less severely affected individuals or those with milder, atypical variants of RTT in which speech is preserved (Neul, 2012). *Mecp2*-null mutant mice, as a mouse model of RTT, largely recapitulate the phenotypes and gender differences in severity observed in individuals with RTT (Lombardi et al., 2015). Female mice heterozygous for a *Mecp2*-null allele (*Mecp2*^-/+^) display a delayed onset of neurological and behavioral deficits at approximately 4 months of age or older, whereas male mice hemizygous for the null allele (*Mecp2*^-/*y*^) are more severely affected, with more rapid regression at approximately 3–4 weeks of age and approximately 50% dying by 8–10 weeks of age.

Studies on postmortem samples from RTT individuals and fixed brains of RTT model mice reported lower spine density of pyramidal neurons in the cortex and hippocampus (Belichenko et al., 1994; Chapleau et al., 2009; Xu et al., 2014). An *in vivo* time-lapse two-photon imaging study revealed defects in short-term spine dynamics in *Mecp2*^-/*y*^ mice (Landi et al., 2011). Densities of spines and filopodia were reduced, and changes in spine length and head volume measured at 5 min intervals for 1 h were smaller in the apical dendrites of L5 pyramidal neurons in somatosensory cortex at P25–26, when the neurological deficits in these mice begin to emerge. In contrast, the short-term spine motility of *Mecp2*^-/*y*^ mice was not different from wild-type mice at P40 or older, when the connectivity within the primary somatosensory cortex was considered mature, although the reduced spine density persisted. A subcutaneous injection of insulin-like growth factor-1 (IGF-1) 24 h prior to the imaging rescued the defects in short-term spine dynamics without ameliorating the reduced spine density. These results indicate that the deficits in structural plasticity of dendritic spines are present at the onset of neurological abnormalities and suggest that pharmacological treatment with IGF-1 during a certain time window in postnatal development may be beneficial for treating RTT.

Although MECP2 is expressed in both principal neurons and interneurons, the excitatory and inhibitory synaptic pathophysiology and circuit dysfunction resulting from loss of MECP2 *in vivo* are poorly understood. Two-photon calcium imaging of the visual cortex of *Mecp2*^-/*y*^ mice has revealed that reduced visually driven excitatory and inhibitory conductances of pyramidal neurons lead to circuit-wide reductions in response reliability, selectivity and signal-to-noise ratios in these cells under both anesthetized and awake conditions (Banerjee et al., 2016). Such cortical processing deficits were recapitulated by *Mecp2* deletion specific to parvalbumin-expressing (PV^+^) interneurons but not somatostatin-expressing (SOM^+^) interneurons and were ameliorated by 2 weeks of daily systemic injections of IGF-1. Moreover, intrinsic-signal optical imaging revealed a role of MECP2 in PV^+^ interneurons in cortical plasticity. Ocular dominance (OD) plasticity, the change in responsiveness of the binocular visual cortex to the eyes after brief monocular deprivation of vision, is a form of visual cortical plasticity whose sensitivity is greatest at approximately 4 weeks of age in mice (Sato and Stryker, 2008). Female mice with heterozygous PV^+^ interneuron-specific *Mecp2* deletion exhibit abnormally prolonged adult OD plasticity, which is a hallmark of reduced firing of PV^+^ interneurons (Kuhlman et al., 2013). This is similar to the previous findings by the same group that female symptomatic *Mecp2*^-/+^ mice exhibited prolonged adult OD plasticity at P60, and this abnormal plasticity was corrected by daily systemic injections of the active tripeptide fragment of IGF-1 or of full-length IGF-1 (Tropea et al., 2009; Castro et al., 2014). Banerjee et al. (2016) further demonstrated that *Mecp2*^-/*y*^ mice exhibit reduced expression of the cation-chloride cotransporter KCC2 and altered GABA reversal potential in pyramidal neurons. Moreover, IGF-1 treatment of these mice restored KCC2 expression in addition to PV^+^ interneuron and pyramidal neuron responses, providing a mechanistic basis for the action of IGF-1. Together, these results demonstrate that loss of MECP2 in the brain alters both excitation and inhibition via multiple mechanisms, and MECP2 deletion in a specific cell type critically contributes to circuit-wide deficits in RTT.

IGF-1 is a growth factor important for brain development and activates multiple intracellular signaling pathways, such as the phosphoinositide 3-kinase (PI3K)-Akt and mitogen-activated protein kinase (MAPK) pathways. IGF-1 mRNA expression is decreased in the cerebellum of *Mecp2*^-/*y*^ mice (Mellios et al., 2014), and IGF-1 protein levels are reduced in the serum of *Mecp2*^-/*y*^ and *Mecp2*^-/+^ mice (Castro et al., 2014; Mellios et al., 2014). Systemically administered IGF-1 or its tripeptide fragment crosses the blood-brain barrier and improves a wide range of behavioral and cellular phenotypes in *Mecp2*^-/*y*^ and *Mecp2*^-/+^ mice (Tropea et al., 2009; Castro et al., 2014). IGF-1 treatment also increases the number of glutamatergic synapses in neurons derived from induced pluripotent stem cells from individuals with RTT (Marchetto et al., 2010). An open-label phase I clinical trial of recombinant human IGF-1 (mecasermin) in girls with RTT indicated good tolerance and improvement of certain breathing and behavioral abnormalities (Khwaja et al., 2014).

A recent study using VSD for imaging of the primary somatosensory cortex of *Mecp2*^-/*y*^ mice revealed weaker and more diffuse whisker-evoked responses at 1–2 months of age (Lee L.J. et al., 2017). This functional deficit was accompanied by reduced complexity of thalamocortical axon terminals at P7 as well as reduced dendritic complexity and spine density of L4 spiny stellate neurons at P30–45, as examined in fixed sections. These findings suggest that similar functional and anatomical defects may underlie sensorimotor behavioral abnormalities in RTT, such as hand-to-mouth stereotypies.

## *In Vivo* Optical Imaging of as Model Mice

Angelman syndrome is a relatively rare NDD, afflicting only 1 in 10,000-40,000 people (Clayton-Smith and Laan, 2003), and was first documented by the British pediatrician Harry Angelman (Angelman, 1965). Individuals with AS display a wide variety of symptoms, including severe developmental delay, speech impairment, ataxia, seizures and abnormal EEG, and behavioral uniqueness, such as frequent laughter and hand flapping (Williams et al., 2006). This disorder is caused by genetic abnormalities affecting the maternal expression of the ubiquitin E3 ligase gene *UBE3A*, resulting in marked loss of function in the brain because the expression of this gene on the paternal chromosome is silenced by genomic imprinting (Sato, 2017). Consistent with a causal role for AS, maternal *Ube3a*-deficient (*Ube3a*^*m*-/*p*+^) mice exhibit behavioral deficits analogous to those of human AS. UBE3A reportedly ubiquitinates several substrate proteins (Sell and Margolis, 2015), but none of them has been directly linked to symptoms observed in individuals with AS.

Although AS is reported to have a high comorbidity with ASD, this must be interpreted with caution because fulfillment of diagnostic criteria for ASD by children with AS could be better explained by severe developmental delay and language impairment in AS rather than by the specific deficits in social and communicative skills typically seen in ASD (Trillingsgaard and Østergaard, 2004; Hogart et al., 2010). Instead, accumulating evidence suggests that while loss of UBE3A in the brain leads to AS, abnormally elevated expression levels or activity of UBE3A may contribute to the pathogenesis of ASD. Maternal duplication or triplication of the chromosomal region 15q11–13, which encompasses *UBE3A*, is one of the most frequent genetic causes of ASD (Takumi and Tamada, 2018; see also the next section). Mice carrying a triple dose of *Ube3a* display an ASD-like phenotype (Smith et al., 2011; Krishnan et al., 2017). In addition, an ASD-linked mutation of *UBE3A* leads to enhanced UBE3A activity through disruption of protein kinase A-mediated phosphorylation control (Yi et al., 2015). These findings suggest a dual role of UBE3A in the pathogenesis of AS and ASD in a gene dosage- and ubiquitin ligase activity-dependent manner.

A postmortem histological study of individuals with AS demonstrated reduced dendritic spine density on L3 and L5 pyramidal neurons of the visual cortex (Jay et al., 1991). AS model mice exhibited reduced dendritic spine density not only on basal dendrites of L5 pyramidal neurons in binocular visual cortex (Sato and Stryker, 2010) but also on L2/3 pyramidal neurons in visual cortex (Yashiro et al., 2009) and on secondary apical dendrites of L3–5 cortical pyramidal neurons (Dindot et al., 2008). To elucidate the mechanisms behind this reduced spine density, GFP-labeled spines on L5 pyramidal neurons in the visual cortex of AS model mice were imaged by *in vivo* time-lapse two-photon microscopy (Kim et al., 2016). This study found that in juveniles, spine formation was normal but elimination was enhanced. Moreover, when AS mice were raised in darkness, spine density and turnover were indistinguishable from those in wild-type mice. Thus, the absence of UBE3A function appears to impair experience-driven spine maintenance, which may explain the decreased excitatory synaptic connectivity in this AS mouse model (Wallace et al., 2012).

To elucidate specific functions of UBE3A in brain development and maturation, intrinsic-signal optical imaging was used to examine experience-dependent plasticity of the visual cortex in juvenile and mature AS model mice (Sato and Stryker, 2010). OD plasticity was impaired in juvenile AS mice (P25-28) compared to age-matched wild-type mice. Moreover, while monocular visual deprivation in mature AS mice (P33-37) elicited plasticity of a magnitude similar to that in wild-type mice, the nature of the plasticity was reminiscent of that during the juvenile period (i.e., weakening of the deprived-eye response). In contrast, mature wild-type mice exhibited strengthening of the non-deprived-eye response. Thus, this functional imaging study reveals that UBE3A is indispensable for adaptability and maturation of neuronal circuits in the cortex.

## *In Vivo* Optical Imaging of ASD Model Mice

Autism spectrum disorders are etiologically heterogeneous early-onset neuropsychiatric disorders but nonetheless typically exhibit three core symptoms: social deficits, language impairment, and restricted and repetitive patterns of behavior. In most cases, ASD is accompanied by various co-morbid symptoms, such as seizures, sleep disorders, hyperactivity, and anxiety. Abnormal responses to sensory stimuli are also common in ASD. ASD is approximately four times more frequent in males, with a reported frequency of 1 in 42 compared to only 1 in 189 females (Christensen et al., 2016). Although the causes are unknown for the majority of cases, a genetic contribution to ASD susceptibility is strongly supported by family and twin studies. While many single genes have been associated with ASD, symptom heterogeneity suggests multiple genetic abnormalities. Indeed, copy number variants (CNVs) ranging from kilobases to megabases (Mb) and produced by deletion or duplication of chromosomal fragments have recently been implicated in a variety of disorders, including ASD (Takumi and Tamada, 2018). Recent studies report that 10-20% of ASD cases can be ascribed to CNVs, whereas only 5-10% of cases may be due to coding-sequence mutations in genes expressed in the brain (Huguet et al., 2013). Among several CNVs associated with ASD, the 15q11-13 duplication, which causes an NDD often called 15q duplication syndrome, is one of the most frequent, found in 0.25% of ASD cases (Pinto et al., 2014). Several CNV mouse models have been generated by chromosome engineering (Takumi and Tamada, 2018), and collectively, these models are thought to better reflect the genetic and phenotypic heterogeneity of ASD than single-gene KO mouse models. For instance, mice that mimic the paternally inherited 15q11-13 duplication by 6.3-Mb duplication of the syntenic region of mouse chromosome 7 (15q dup mice) display ASD-like behavioral phenotypes, including impaired social interaction, abnormal ultrasonic vocalization, and behavioral inflexibility (Nakatani et al., 2009).

Postmortem studies of individuals with non-syndromic ASD have so far mostly focused on global changes in neuronal cytoarchitecture and number, while relatively few have examined spine abnormalities (Martínez-Cerdeño, 2017; Varghese et al., 2017). An early qualitative study documented reduced spine density on apical dendrites of cortical pyramidal neurons (Williams et al., 1980). A more recent quantitative Golgi-impregnation study on the superficial and deep cortical layers of the frontal, temporal, and parietal lobes found greater spine densities, primarily on L2 pyramidal neurons of each cortical area and L5 pyramidal neurons of the temporal lobe, in individuals with ASD (but without FXS) compared to age-matched controls (Hutsler and Zhang, 2010). In younger ASD cases, increased spine density was also reported on basal dendrites of L5 pyramidal neurons of the temporal lobe (Tang et al., 2014) and on dendrites of principal neurons in the lateral nucleus of the amygdala (Weir et al., 2018).

The spine dynamics of 15q dup mice were examined by *in vivo* two-photon time-lapse imaging (Isshiki et al., 2014). This study specifically labeled subsets of excitatory and inhibitory postsynaptic spines on L2/3 cortical pyramidal neurons by expressing the GFP-tagged postsynaptic marker proteins PSD-95-GFP and gephyrin-GFP, respectively, while dendrites and all spines were filled with the red fluorescent protein DsRed2. These 15q dup mice showed normal spine density but enhanced turnover rates of PSD-95-GFP-labeled spines in somatosensory and anterior frontal cortices. In contrast, gephyrin-GFP-labeled spines were unaffected. Furthermore, altered sensory experience did not alter the elevated basal spine formation rate in the somatosensory cortex of 15q dup mice. Chessboard-like whisker trimming, which increased the formation rate but not the elimination rate of gephyrin-GFP-negative spines in wild-type mice, did not affect the formation rate in 15q dup mice, as it was already comparable to the enhanced spine formation of wild-type mice after whisker trimming. These results indicate that the basal spine turnover rate is constantly high even in the absence of sensory alterations and suggest that the neurons in 15q dup mice lack the capacity to remodel neuronal connectivity in response to new sensory experience. Similar overall spine defects were also observed in another ASD mouse model, neuroligin-3 R451C point mutant mice. These results suggest that ASD is associated with selective impairments of reorganization of PSD-95-containing excitatory synapses receiving intracortical afferents.

Interestingly, the dynamics of gephyrin-GFP clusters on spines remained unchanged, but those on dendritic shafts showed enhanced dynamics in 15q dup mice. Dynamic inhibitory synapses and remodeled spines are clustered close to each other on dendrites, and the occurrence of clustered changes is influenced by sensory experience (Chen et al., 2012; Wefelmeyer et al., 2016). Further, blockade of GABA_A_ receptors elicits increased spine elimination (Chen, 2014), suggesting that altered GABA inhibition may underlie the enhanced spine turnover in 15q dup mice.

Given that 15q dup mice show high spine turnover rates regardless of alterations in sensory experience, this may reflect that sensory-evoked activity is abnormal in the cortex of these mice. A macroscopic calcium imaging study reported that 15q dup mice exhibited broader whisker-evoked response areas in the primary somatosensory cortex (Nakai et al., 2017). Further anatomical and electrophysiological analyses revealed fewer inhibitory synapses and concomitant hyperexcitability of pyramidal neurons, suggesting that the impaired sensory tuning is a consequence of reduced cortical inhibition, since inhibition is known to sharpen the whisker-evoked response by suppressing the responses of the surrounding areas (Foeller et al., 2005; Isaacson and Scanziani, 2011). Although the full mechanisms linking the enhanced spine turnover and the altered sensory circuit function remain to be understood, subsequent analyses revealed that serotonin is a key molecule in the pathophysiology of ASD. The 15q dup mice show reduced brain serotonin levels and decreased size, excitatory drive, and glucose metabolism of the dorsal raphe nucleus, which contains a large proportion of serotonin neurons innervating the cortical forebrain (Tamada et al., 2010; Ellegood et al., 2015; Nakai et al., 2017). Consistent with hypofunction of the serotonergic system in ASD, restoration of brain serotonin levels during the early postnatal period by administration of the selective serotonin reuptake inhibitor (SSRI) fluoxetine alleviated impaired inhibitory synaptic function at 2-3 weeks of age by reinstating the normal mIPSC frequency without affecting amplitude, implying that restoration of inhibition was mediated by an increased number of inhibitory synapses rather than by enhanced strength of individual synapses (Nakai et al., 2017). Moreover, fluoxetine improved a subset of social behavioral defects in both young and adult 15q dup mice. While rescue by fluoxetine is encouraging, it remains to be elucidated whether reduced serotonin also contributes to the ethology of other ASD mouse models. In humans, maternal 15q duplication is a more frequent cause of ASD than paternal duplication. However, ASD-like behavior and reduced brain serotonin are observed in paternal but not maternal 15q duplication mice, implying that susceptibility to serotonergic hypofunction is determined by species- and parent-of-origin-specific genetic mechanisms.

The BTBR T+tf/J inbred mouse strain is considered a robust mouse model of idiopathic ASD, displaying impaired social interactions, communication deficits, and repetitive self-grooming compared to the commonly used C57BL/6 inbred strain (McFarlane et al., 2008; Scattoni et al., 2008). The insular cortex serves to combine sensory, emotional, and cognitive inputs from other brain networks, and aberrant insular connectivity and activation have been reported in ASD (Di Martino et al., 2009; Uddin and Menon, 2009; Ebisch et al., 2011). Intrinsic-signal optical imaging of the insular cortex revealed impaired multisensory integration in the BTBR T+tf/J mouse model (Gogolla et al., 2014). Specifically, these mice exhibited exaggerated auditory responses and lacked enhancement of insular responses to concurrent audio-tactile stimuli. The impaired postnatal maturation of integrated responses reflected weakened cortical GABA circuits. Consistent with deficient GABA inhibition in insular circuit dysfunction, transient pharmacological enhancement of inhibition by diazepam early in life rescued these deficits in the adult. Moreover, impaired insular multisensory integration has been observed across different monogenic ASD mouse models, including *Shank3* KO mice and *Mecp2* KO mice. These models also exhibit altered excitatory-inhibitory (E/I) balance, suggesting that optimal E/I balance is indispensable for proper maturation of circuits mediating multisensory integration.

Several genes associated with ASD, such as those encoding neuroligins, neurexins, and Shanks, are involved in the formation and function of synapses (Huguet et al., 2013). *MDGA2* (MAM domain-containing glycosylphosphatidylinositol anchor 2) is a recently identified ASD susceptibility gene that encodes a protein that negatively regulates the synaptogenic activity of neuroligins by suppressing their interaction with neurexins (Bucan et al., 2009; Connor et al., 2016). *Mdga2* haploinsufficient (*Mdga2*^+/-^) mice exhibit an ASD-like behavioral phenotype and increased excitatory synaptic number and function (Connor et al., 2016). VSD imaging of resting-state cortical activity of *Mdga2*^+/-^ mice revealed widespread increases in cortical spontaneous activity, notably in the posterior secondary motor cortex, retrosplenial cortex and primary motor cortex for whiskers and forelimbs (Connor et al., 2016). This technique also demonstrated intrahemispheric cortical functional hyperconnectivity, particularly in lateral cortical areas involving secondary somatosensory cortices and primary auditory cortex (Connor et al., 2016). Functional connectivity analysis using VSD imaging could be comparable to the rsfMRI described above, although VSD imaging is an optical method and can only image the superficial cerebral cortex. The midline cortical regions that displayed enhanced activity in *Mdga2*^+/-^ mice are considered part of a putative mouse equivalent of the so-called default-mode network (Gozzi and Schwarz, 2016), a distributed intrinsic resting-state brain network implicated in various cognitive processes and brain disorders (Raichle, 2015). Indeed, functional brain hyper- or hypoconnectivity has also been observed in fMRI of ASD children (Ecker et al., 2015).

## *In Vivo* Optical Imaging of 22q11.2DS Model Mice

22q11.2DS (also known as vero-cardio-facial syndrome or DiGeorge syndrome) is a complex neurogenetic disorder caused by a hemizygous microdeletion of 1.5–3 Mb on chromosome 22 and is one of the most common CNVs with a frequency of approximately 1 in 2,000–4,000 live births (Kobrynski and Sullivan, 2007; Karayiorgou et al., 2010). The clinical features of this disorder include cleft palate, hypocalcemia, cardiac defects, immune dysfunction, short stature and developmental delays. 22q11.2 deletion is associated with an increased risk for neuropsychiatric disorders. Strikingly, up to one-third of individuals with 22q11.2 deletion develop schizophrenia (SZ), and this accounts for 1–2% of sporadic SZ in the general population (Karayiorgou et al., 2010). In addition, 22q11.2 deletion carriers often meet diagnostic criteria for other disorders such as attention-deficit/hyperactivity disorder (ADHD), anxiety disorder, mood disorder and ASD over their lifetimes, although it remains controversial whether neuropsychiatric diagnoses other than SZ represent non-specific deficits in brain development and function or true genetic pleiotropy of this deletion (Jonas et al., 2014). A mouse model carrying a hemizygous 1.3 Mb-deletion of the syntenic region of mouse orthologous chromosome 16 [*Df(16)^+/-^*] shows reduced dendritic complexity and decreased density of mushroom-shaped spines and excitatory synapses in fixed sections of the hippocampal CA1 area (Mukai et al., 2008). Multiple mouse models of 22q11.2 deletion, including *Df(16)^+/-^*, exhibit SZ-related behavioral phenotypes, including deficits in sensory-motor gating, working memory, and fear memory (Stark et al., 2008; Drew et al., 2011). In addition, *Df(16)^+/-^* mice exhibit impaired social cognition, a symptom commonly observed in SZ and ASD (Piskorowski et al., 2016).

A recent calcium imaging study sought a neural substrate of cognitive deficits by examining hippocampal neural circuit dynamics of awake, behaving *Df(16)^+/-^* mice (Zaremba et al., 2017). Hippocampal pyramidal cells are known to exhibit place cell activity; they are active when an animal visits specific locations within an environment. In this study, head-fixed *Df(16)^+/-^* mice under a two-photon microscope were trained to find a reward on a sensory cue-rich treadmill belt, and their hippocampal CA1 neurons labeled by adeno-associated virus (AAV) vector-mediated GCaMP6f expression were imaged though a window implanted after surgical removal of the overlying cortex. *Df(16)^+/-^* mice showed a significant deficit in goal-oriented learning when the environmental context or the reward location was changed, along with reduced day-to-day stability of place cell maps compared to wild-type mice. In addition, they lacked reorganization of place cell maps toward the goal location. These results demonstrate that hippocampal neuronal ensemble dynamics that support cognitive flexibility are impaired in a mouse model of 22q11.2 DS.

## Serotonin-Mediated E/I Rebalancing as a Potential Therapeutic Target for ASD

An E/I imbalance as a key factor in ASD etiology was proposed more than a decade ago (Rubenstein and Merzenich, 2003), and since then, substantial evidence has accumulated in support of this hypothesis. Postmortem studies of individuals with ASD demonstrate downregulation of markers for GABA inhibition (Oblak et al., 2009; Blatt and Fatemi, 2011), and many ASD mouse models display altered E/I balance through multiple mechanisms (Lee E. et al., 2017; Bozzi et al., 2018). Moreover, pharmacological compounds that modulate the GABA system have been tested for therapeutic efficacy in mouse models, and clinical trials are currently ongoing (Braat and Kooy, 2015). However, a recent theoretical study proposed that a simple unidimensional E/I imbalance model cannot fully account for the aberrant neural circuit activity in *Fmr1* KO mice (Gonçalves et al., 2013; O’Donnell et al., 2017), implying that restoration of E/I balance by direct modulation of GABA signaling alone may be insufficient for some symptoms or forms of ASD.

The serotonin system plays multiple roles in brain development and function (Lesch and Waider, 2012). Positron emission tomography studies revealed that the serotonin synthesis capacity is reduced in the brains of children with ASD (Chugani et al., 1999; Chandana et al., 2005). Moreover, elevated whole-blood serotonin, which may reflect increased platelet serotonin uptake as shown in mice harboring an ASD-associated gain-of-function mutation in the gene encoding serotonin transporter (SERT Ala56 mice; Veenstra-VanderWeele et al., 2012), is found in more than 25% of affected children, suggesting hyperserotonemia as a biomarker for ASD (Muller et al., 2016). Although a concomitant increase in brain serotonin clearance in SERT Ala56 mice may lead to decreased synaptic serotonin availability and compensatory serotonin receptor hypersensitivity (Veenstra-VanderWeele et al., 2012), it remains to be investigated whether hyperserotonemia is correlated with reduced brain serotonin levels in ASD. SERT Ala56 mice also show behavioral deficits in multisensory processing (Siemann et al., 2017), implying that abnormal serotonin levels may be involved in altered multisensory processing in ASD. Serotonin modulates the strengths of excitatory and/or inhibitory synapses in a serotonin receptor subtype-specific manner (Ciranna, 2006; Lesch and Waider, 2012) and enhances sensory representation and discrimination by adjusting the relative strengths of distinct input pathways (Stutzmann et al., 1998; Kapoor et al., 2016; Tang and Trussell, 2017). Serotonergic modulation has therefore been proposed to be a suitable target for restoring E/I balance and sensory processing that are altered in ASD.

In light of this putative role of serotonin in the pathophysiology of ASD, several clinical studies have investigated the therapeutic effects of SSRIs, particularly on repetitive behaviors, as SSRIs are the established first-line treatment for obsessive–compulsive disorder (OCD) (Kellner, 2010). However, the results thus far are inconsistent, and there is currently no positive consensus on their efficacy according to recent systematic reviews (McPheeters et al., 2011; Doyle and McDougle, 2012; Williams et al., 2013). There have been several published placebo-controlled SSRI studies (McDougle et al., 1996; Hollander et al., 2005, 2012; Sugie et al., 2005; King et al., 2009). Among them, a single-site double-blind placebo-controlled 8-week crossover study of 39 children and adolescents (5-16 years old; 90% ASD and 10% Asperger) found that fluoxetine (2.4-20 mg/d) was superior to placebo in decreasing repetitive behaviors, with no significant difference in occurrence of adverse effects (Hollander et al., 2005). Similarly, a double-blind placebo-controlled 12-week parallel study of 37 adults [18-60 years old; 65% Asperger, 32% ASD and 3% pervasive developmental disorder not otherwise specified (PDD-NOS)] found a significantly greater reduction in repetitive behaviors in the fluoxetine-treated group (10-80 mg/d) than in the placebo-treated group, with only mild and moderate side effects (Hollander et al., 2012). However, preliminary results from a larger, multi-site, double-blind placebo-controlled study of 158 children (5-7 years old) have found that a novel fluoxetine formulation is no more effective than placebo for the treatment of repetitive behaviors (Autism Speaks, 2009). In addition, another multi-site, double-blind placebo-controlled study of the SSRI citalopram (2.5-20 mg/d), including 149 children and adolescence (5-17 years old; including ASD, Asperger and PDD-NOS with unknown percentages), reported no significant difference between citalopram and placebo groups in measures of repetitive behavior (King et al., 2009). Moreover, adverse events, including increased energy, impulsiveness, decreased concentration, hyperactivity, stereotypy, diarrhea, insomnia, and itchy dry skin (pruritus), were significantly more frequent in the citalopram group (King et al., 2009).

These discordant results may be due to the varying degrees of selectivity for the serotonin transporter over other actions and the different pharmacokinetic and pharmacodynamic profiles of SSRIs used. Furthermore, the pharmacological properties of a single SSRI can also change during development. Currently available SSRIs may not be effective for ASD in general because of the extreme heterogeneity of ASD etiology and the diversity of serotonin signaling systems. It is possible, however, that SSRIs may benefit certain forms of ASD. This outcome highlights the necessity of personalized medicine for ASD treatment, a strategy considered routine for cancer treatment. As discussed above, 15q11-13 duplication is a relatively common genetic abnormality in ASD, but the absolute frequency is rather low. In addition, administration of SSRIs to ASD individuals without reduced brain serotonin levels will not only be ineffective but also will likely cause adverse effects. Pre-treatment genotyping and assessment of brain serotonin synthesis capacity are thus recommended to identify cases potentially treatable with SSRIs. Serotonin receptors include several classes and numerous subtypes, and these subtypes are differentially expressed among neuronal types and brain regions, making it difficult to predict the effects of SSRI-mediated serotonin increases in individuals with ASD. Indeed, while postnatal fluoxetine treatment improved impaired social behavior in 15q dup mice, the same treatment increased anxiety-like behavior in these mice and even impaired reversal spatial learning and exploratory behavior in wild-type mice (Nakai et al., 2017). These observations imply that despite the relatively well-established safety of SSRIs as antidepressants in adults, late-emerging adverse effects of postnatal SSRI treatment require further investigation by longitudinal assessment. Administration of SSRIs to depressed women during pregnancy or after delivery is known to increase serotonin levels in the fetus via the placenta or in newborns via breast milk. Although still controversial, accumulating evidence suggests that such perinatal SSRI exposure is a potential risk for a wide range of symptoms, including ASD, and placental and lactational transfer of SSRIs leads to abnormal behavior and various structural and functional alterations of the brain in rodents (Kinast et al., 2013). Furthermore, SSRIs are less well tolerated in children than adults, and the FDA has not approved SSRIs for OCD in children younger than 6-8 years (Williams et al., 2013). In sum, there are many issues to be resolved before safe and effective pharmacological interventions for restoration of brain serotonin levels in children with ASD are possible. Further elucidation of developmental changes in serotonin subsystems and downstream mechanisms underlying different ASD symptom domains is needed for the development of more-specific pharmacological therapies.

## Conclusion and Outlook: Lessons From *In Vivo* Optical Imaging

*In vivo* optical imaging is a powerful technique for investigating brain structure and function in living animals at the circuit, cellular, and synaptic levels, and it will thus continue to be widely applied to new NDD mouse models, since the range of its application has so far been limited to several currently available mouse models among many NDDs. Further, the spatiotemporal resolution and modality of *in vivo* optical imaging are expected to increase with new developments in optics, microscopes, and fluorescent indicators. These findings and implications lead us to the following conclusions.

**FIGURE 2:**
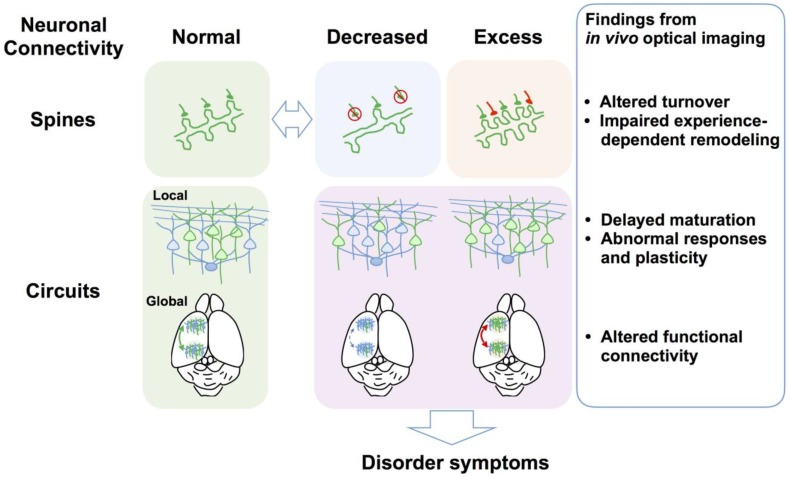
Defects of spine dynamics and circuit function as common pathophysiological underpinnings of NDDs. Multiple NDD model mice and postmortem brains from individuals with NDDs exhibit reduced (e.g., RTT and AS) or increased spine density (e.g., FXS), which is a hallmark of abnormal neuronal connectivity. Evidence from *in vivo* optical imaging of NDD mouse models suggests that altered spine turnover (i.e., formation and elimination) and impaired experience-dependent remodeling are putative common phenotypes across NDDs. At the local circuit level, *in vivo* optical imaging has revealed abnormal sensory responses and plasticity and defects of circuit maturation in common, although causal links between observed aberrant neuronal connectivity and impaired local circuit function at the gross level remain to be elucidated. *In vivo* optical imaging has recently demonstrated altered functional connectivity between different brain areas at the global circuit level. Findings obtained using *in vivo* optical imaging at multiple levels thus greatly advance the understanding of the neural circuit bases for neurological and behavioral symptoms of NDDs.

(1) *In vivo* time-lapse imaging of dendritic spines of NDD mouse models can illuminate dynamic turnover processes that cannot be revealed by examination of fixed brains. In most cases, spine density imaged *in vivo* is consistent with observations in fixed tissues, but it can also differ depending on factors such as age, genetic background, species, cortical area, cortical layer, cell type, labeling technique, and imaging method. Technically, *in vivo* imaging can better visualize dendrites close to the brain surface, such as apical dendritic tufts in L1, so the results accumulated thus far are not necessarily applicable to deeper cortical layers and other brain areas. Studies have also demonstrated that the direction of spine density change differs according to the disorder model, with different models exhibiting increased (FXS), reduced (RTT and AS), or unchanged (15q dup) spine density. However, altered spine turnover rate and impaired experience-dependent remodeling appear to be common phenotypes across multiple disorder models (**Figure 2**).

(2) *In vivo* functional imaging has revealed that multiple NDD model mice exhibit abnormal cortical sensory responses, such as broader, fast-spreading, or undifferentiated cortical responses, suggesting abnormal cortical representations of external stimuli. Neuronal hyperexcitability and associated behavioral phenotypes such as seizures in some mouse models suggest that altered E/I balance underlies these abnormal sensory responses. *In vivo* calcium imaging can simultaneously record the activity of a large, dense population of neurons. The large datasets obtained with this technique are useful for testing the validity of theoretical models, and such a combined optical and theoretical approach may yield alternative theoretical models regarding the etiology of disorders (O’Donnell et al., 2017).
(3) *In vivo* optical imaging can also guide the development of new pharmacological interventions for NDDs (Tropea et al., 2009; Landi et al., 2011; Castro et al., 2014; Gogolla et al., 2014; Zhang et al., 2014; Banerjee et al., 2016; Nagaoka et al., 2016; Nakai et al., 2017). *In vivo* optical imaging is particularly useful for identifying and validating potential therapeutic targets when combined with anatomical, molecular, electrophysiological, and behavioral analyses. The findings obtained from such studies are expected to provide foundational support for clinical studies on improved therapeutic strategies.
(4) One of the strengths of *in vivo* optical imaging is the ability to visualize specific subtypes of neurons and synapses through differential labeling (Isshiki et al., 2014). However, this strategy has not yet been fully exploited in studies on mouse models of NDDs, so substantial advances in our understanding of cell- and synapse-specific defects are expected in the years to come. This approach is also very important to fill the gap between spine defects and abnormal circuit function because circuit functions emerge from complex neuronal networks that contain different types of synapses, including those made by local excitatory and inhibitory neurons as well as long-range connections from distant areas (**Figure 2**).
(5) Due to optical limitations, most *in vivo* imaging studies have focused on the cerebral cortex. However, subcortical brain areas should also be imaged in future studies because NDDs affect these deep brain regions as well (Zaremba et al., 2017). Recent advances in deep brain imaging techniques, such as targeted cortical excavation and microendoscopy using gradient refractive-index lenses (Ji et al., 2016; Sato et al., 2016, 2017b), may allow such applications. In addition, most experiments discussed here imaged sensory responses under anesthesia. The use of awake head-fixed animals (Banerjee et al., 2016; He et al., 2017; Sato et al., 2017a; Zaremba et al., 2017) and miniature head-mounted fluorescence microscopes attached to freely moving animals (Ghosh et al., 2011; Cai et al., 2016) will enable imaging of neural circuit activity while mice perform cognitive tasks relevant to the disorder of interest.

Pathophysiological changes at the molecular and circuit levels are complex even for monogenic NDDs, so non-syndromic and idiopathic conditions present enormous challenges. However, elucidating such changes is a necessary step toward the development of safe and effective therapies for these lifelong conditions. *In vivo* optical imaging of mouse models of NDDs will continue to contribute to this endeavor by providing evidence for dysfunction in the living brain.

## Author Contributions

NN and MS wrote the manuscript with input from TT and JN.

## Conflict of Interest Statement

The authors declare that the research was conducted in the absence of any commercial or financial relationships that could be construed as a potential conflict of interest.
